# Gels, Aerogels and Hydrogels: A Challenge for the Cellulose-Based Product Industries

**DOI:** 10.3390/gels8080497

**Published:** 2022-08-10

**Authors:** Bogdan-Marian Tofanica, Dan Belosinschi, Irina Volf

**Affiliations:** 1 Laboratory for Complex and Integrated Processing of Biomass Resources, Faculty of Chemical Engineering and Environmental Protection, “Gheorghe Asachi” Technical University of Iasi, 73, Prof. Dr. Docent D. Mangeron Boulevard, 700050 Iasi, Romania; 2 Département de Chimie-Biologie/Biologie Medicale, Université du Québec à Trois-Rivières, Trois-Rivieres, QC G8Z 4M3, Canada

**Keywords:** cellulose, polymers, gels, aerogels, hydrogels

## Abstract

During recent decades, the interest in renewable, biodegradable, non-fossil materials has been exponentially increasing. Thus, cellulose and cellulose-derived products have been extensively considered for a wide variety of new potential uses. Due to the sustainability of cellulosic raw materials and their excellent properties, the use and modification of cellulose-based materials can be versatile in the material science and technology community. In this featured article, the fundamentals and background of cellulose-based gels are presented, and approaches, prospects and developments in the field, including their potential future applications, are discussed.

## 1. Introduction

According to the latest version of The International Union of Pure and Applied Chemistry (IUPAC) Gold Book [1], gels are defined as non-fluid colloidal network or polymer network that is expanded throughout its whole volume by a fluid. Additionally, the Polymer Science Dictionary [2], describe gels as crosslinked polymers and their swollen matters with three-dimensional network structures that are insoluble in any solvents (Appendix A). A gel consists of a solid three-dimensional network that forms the structure and a medium within. Although it is possible for the medium to be a gas, it can also to be a fluid. Generally speaking, there are aerogels, which use air as the medium, and hydrogels when a liquid is the medium for a gel [3].

Among the polymers that can form gels, cellulose and emerging cellulose-based nanomaterials have recently become of increased interest as a sustainable and renewable material that has the potential to produce low-cost and high-performance gels [4]. Cellulose in various forms: nanocrystals (CNC), nanofibrils (CNF), nanocrystalline (NCC), nanowhiskers (CNW), nanofibrillated cellulose (NFC) and microfibrillated cellulose (MFC), can be isolated from naturally rich cellulosic sources such as wood, cotton, annual plants, tunicates, algae and bacteria by controlled mechanical or chemical treatment, or by a combination of the two.These cellulose-based resources that have at least one dimension on the nanoscale (lower than 100 nm) are being considered for many potential uses in a wide range of biomedical, energy, and separation applications and in cosmetic and food products.

The characteristics of cellulose gels, such as molecular mass, degree of crystallinity, mechanical performance, thermal stability and physicochemical properties on one side, and structural properties, extraction processes used in their production and pretreatment and post-treatment processes on the other side, are determined by the raw materials from which they derived. Therefore, cellulose-based gels are divided into three categories determined by the source from which their polymer is derived: natural cellulose gels, regenerated cellulose gels and cellulose derivate gels.

The purpose of the present manuscript is not to provide another review, but, considering the important developments in the use of cellulose-based gels, to build on the accumulated knowledge in this area and to deliver a synthetic explanation of cellulosic gels including: (i) the preparation of cellulose-based gels; (ii) the properties of cellulose-based gels; and finally (iii) the applications of cellulosic gels in common fields of application.

## 2. Preparation of Cellulose-Based Gels

Cellulose is considered to be the most abundant natural polymer, being mainly found in the cell walls of land plants. Native cellulose is found in both hardwoods and softwoods (as their main structural component—about 50% cellulose) and cotton (about 95%), which are the major sources for conversion to a wide variety of useful products, including: materials (fibers for pulp and paper, textiles, cardboard, construction panels, etc.), chemicals (plastics, films, emulsifiers, thickening additives in food, feed, cosmetics, etc.) and energy (direct burning, conversion to fuels, etc.).

**Figure 1:**
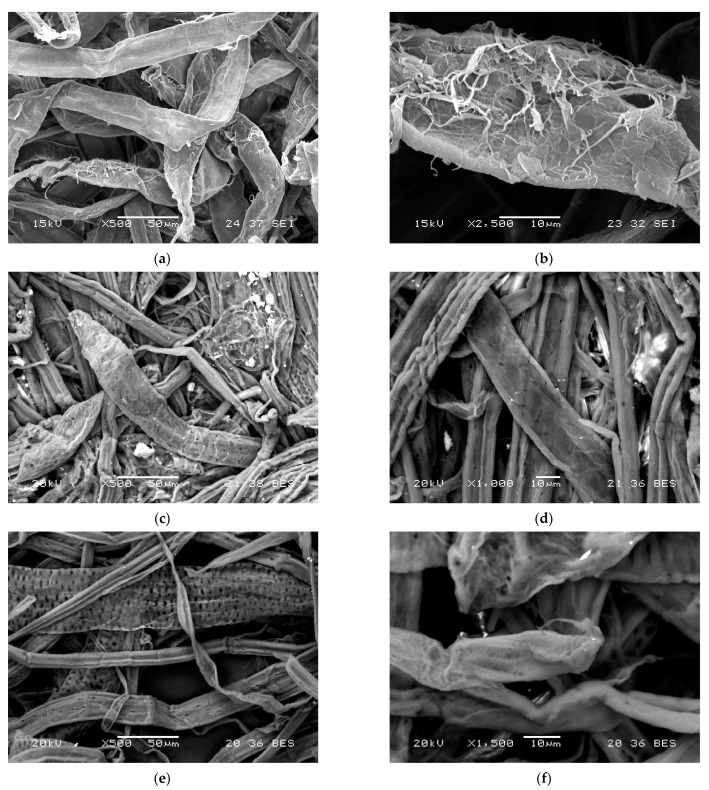
Scanning electron microscopy of plant cell walls at different magnifications. (**a**) General view of softwood fibers (magnification 500×). (**b**) Broken ends of softwood fibers (magnification 2500×). (**c**) Cellular elements in rapeseed pulp (magnification 550×). (**d**) Fibers in rapeseed pulp (magnification 1000×). (**e**) Perforations in corn fibers (magnification 500×). (**f**) Fibers in corn pulp (magnification 1500×).

Since cellulose is chemically a very stable water-insoluble polysaccharide, the production of aerogels from cellulose requires a technology or processing route to break down the plant cell walls into building elements, as seen in Figure 1 (best without degradation of the fiber or reduction of the degree of polymerization) and then to assemble them into an appropriate low-density, open porous material that can be dried to obtain a 3D structure using supercritical carbon dioxide or lyophilization, organic solvent-mediated freeze drying at ambient pressure or low vacuum [5,6,7,8,9].

Although cellulose exhibits hydrophilic and hygroscopic properties, due to inter- and intramolecular hydrogen bonding between the hydroxyl groups in the macromolecular chains, it is insoluble in water and most organic solvents. There are two ways to dissolve cellulose: dissolving it in a solvent without any further modification or by derivatization. Three dissolving agents are widely used for aerogels [10]:

- NaOH or LiOH water solutions with additions of small amounts of urea, thiourea, polyethylene glycol (PEG), ZnO and others [11];
- Ionic liquids, most frequently based on N-methylmorpholine-N-oxide (NMMO) with stabilizers against oxidation [12,13];
- Molten salt hydrate, such as ZnCl_2_, Ca(SCN)_2_, LiClO_4_, etc. [14].

Derivatization is a technique used to modify the physical and chemical properties of cellulose and an important route to functionalizing it. Cellulose can be converted to esters or ethers (cellulose acetate, cellulose nitrate, cellulose sulfates, cellulose phosphate, methylcellulose, ethylcellulose, hydroxypropyl cellulose, hydroxypropyl methylcellulose, carboxymethylcellulose, etc.) that are soluble in water and/or typical organic solvents [15].

Cellulose aerogels are mainly made from regenerated cellulose. Regeneration after dissolution to the polymeric level leads to polymeric chains consisting of pure cellulose macromolecules, generally known as type II cellulose, compared with cellulose I, natural cellulose. The best-known route is the viscose process leading to rayon filaments, known as mercerization. Cellulose is soaked below room temperature in a strong alkali solution such that the crystalline structure converts from cellulose I to cellulose II polymorphs. The alkali–cellulose solution is mixed with toxic carbon disulfide to form cellulose xanthate, resulting in a very viscous liquid, viscose, that can be converted back into cellulose by immersion in an acid medium and extruded through a spinneret to make rayon filaments [16].

Cellulose hydrogels can be obtained via physical stabilization or chemical reaction of cellulose, cellulose derivatives or a mixture of the two. In chemically formed hydrogels, covalent interactions are developed between functional groups of the macromolecular chains [17]. The main covalent coupling reactions used are Michael additions, click chemistry reactions, Schiff’s base formation, photo-cross-linking and enzyme-mediated cross-linking. Agents for cross-linking are usually employed to build covalent interactions between macromolecular chains. Physically formed hydrogels are produced by physical interactions, such as hydrogen bonding, Van der Waals forces, electrostatic interactions, chain entanglements and hydrophobic forces, to cross-link molecule chains [18,19].

## 3. Properties of Cellulose-Based Gels

The methods used to characterize cellulose gels are the same as for wood chemistry in classical materials science, as shown in Table 1. However, some specific features of cellulose-based gels should be considered in order to obtain reliable information. On the other hand, considering the large number of parameters used to prepare gels, an adequate comparison is quite challenging. Gels are water-swelling, 3D polymeric networks that have a huge capacity to absorb liquids that can be as much as thousands of times the mass of the polymers themselves. High porosity, high specific surface, high mechanical strength, low density and hydrophilic nature are the main features of cellulose-based gels that have played an essential role in the development of new uses [20].

**Figure 2:**
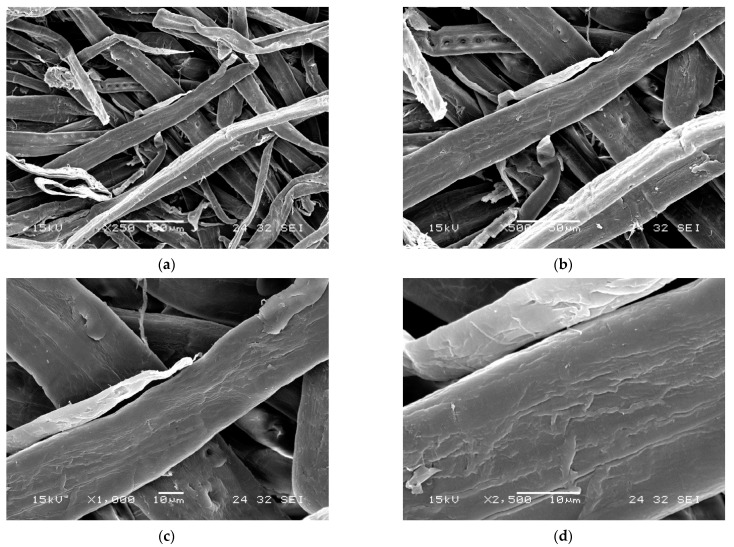
Scanning electron micrographs of gels derived from cellulose-based materials–kraft softwood fibers. (**a**) Magnification 250×. (**b**) Magnification 500×. (**c**) Magnification 1000×. (**d**) Magnification 2500×.

**Electron microscopy.** Scanning electron microscope (SEM) and transmission electron microscope (TEM) are commonly used methods to study the morphology and microstructure of cellulose-based gels, however, they cannot be used to quantify them. As can be seen in Figure 2, the scanning mode methodology with transmitted signal collection help to observe the surface and interior morphology and calculate the specific surface area and pore distribution (micro-, meso- or macro-pores), in order to facilitate characterization of gel textures [21].

**Porosity, specific surface area and density characterization** are important properties of three-dimensional porous materials that are usually determined by measuring sample mass, dimensions and volumes, which are always necessary for the cellulose-based gels to be efficiently used as adsorbents, insulators, catalysts, etc. High porosity, large specific surface area and low density provide high mechanical properties, high adsorption capacity, high impact shock absorption and lightweight characteristics for gel materials [22].

**Mechanical characterization.** The mechanical properties of cellulose-based gels are closely dependent on, and explicable by, the morphology of the gel. Generally, the larger the pores in the gel three-dimensional structure, the lower the mechanical attributes. Furthermore, the porosity of the fabricated gel is dependent on the initial precursor material, methods of preparation, additive materials, fillers and physical conditions. In principle, cellulosic gels demonstrate higher mechanical strengths, such as high modulus, compressive strength, energy absorption capacity, flexibility, etc. [23].

**Rheology.** Rheological parameters of cellulose gels are employed to characterize the types of structural organization found in the system (i.e., association, entanglement and cross-links). These properties are influenced by properties of cellulosic raw materials such as crystallinity, degree of polymerization/molecular mass, gelation properties and the type of process involved in the gel formulation [24].

**Swelling.** The ability to display a quantifiable change in volume in response to external stimuli is an important property of gels. In the case of aerogels, cellulose is readily wetted by water, even when exposed to the atmosphere, and exhibits considerable swelling until saturated once they have absorbed appreciable amounts of water. Chemical modification can change the hygroscopic and hydrophilic behavior of cellulose-based aerogels. In hydrogels, the degree of crosslinking influences the volume permitted for diffusion inside the network and, subsequently, their capacity to take up fluids/water [25].

**Conductivity.** Thermal, electric and sound conductivity are special and exciting properties of gels. Because of high porosity and low density, some aerogels are to be considered insulating materials, while, intuitively, it is clear that hydrogels, hydrophilic and adsorbing in nature, favor conductivity. Molecular modelling can be very helpful in devising new desired properties and new applications [26].

**Chemical/physical characterization.** Various chemical modifications of cellulose gels have been developed to extend their intractable intrinsic properties: esterification, etherification, sulphonation, phosphorylation, oxidation, and polymer grafting have all been applied through the chemical modification of hydroxyl groups.

The presence of functional groups on the cellulosic macromolecular backbone affects all properties. Thus, the physical and chemical characteristics of the gels are adjusted through the modification of functional groups. The presence of functional groups on the cellulose backbone can be characterized easily by ultra-violet-visible spectroscopy, nuclear magnetic resonance, infrared spectrophotometry and mass spectrophotometry [27].

## 4. Applications

Cellulose-based gels are commonly used in many different domains, industries and environmental areas of application: textile, agriculture, horticulture, personal hygiene products, biomedical, pharmaceuticals, etc.

Properties of cellulose-based gels such as viscosity, solubility, porosity, absorbency, permeability, elasticity–flexibility, high water content and high surface area give this class of materials a remarkable array of applications: antimicrobial, antibacterial, antifungal, antiviral, nontoxic, wound dressing, tissue engineering, regenerative medicine, drug delivery, personal care products, barrier and food packaging, pharmaceuticals, biomedicine, etc.

These materials are also being increasingly exploited in smart materials and applications, such as stimuli–response materials, contaminant removal, corrosion inhibitors, delivery of agrochemicals (pesticides, fertilizers, etc.), water treatment and water withholding in desert and arid areas [28].

Furthermore, thanks to cellulose’s chemical reactivity, large number of diverse derivatives, various functionalities, flexible preparation process, and numerous methods of modification, cellulose-based gels are generally multi-functional.

Currently, through efficient methods to tailor gels’ properties, they are mainly used in adsorption and separation, insulation and construction materials, biomedical devices, packaging products, sorbents, environmental remediation, the automotive industry, electronics, sensors and apparel [29,30].

The current analysis will not focus on discussion of niche products or emerging applications related to non-industrial large-scale relevance, including those in polymer/metal particles composites (metal nanoparticle support, magnetic gels, quantum dots, photocatalytic materials, etc), carbon-cellulose gels (catalysts, sensors, proton exchange membrane fuel cell, CO_2_ capture, etc), cellulosic gels in electrical devices and energy storage (sensors, electrodes for batteries, supercapacitors, wearable and portable electronics, electronic paper, optical materials, etc), anti-bacterial/-viral/-microbial/-fungal materials (silver nanoparticles embedded cellulosic gels, etc).

**Biomedical application.** Natural, non-toxic, biodegradable and biocompatible cellulose gels can be used in drug delivery, cell culture, cell therapy, cell biology, biosensors, regenerative medicine, drug development and many other biomedical applications. Cellulose-based aerogels are receiving growing interest in biomedical and pharmaceutical applications due to their porous structure and high surface area, which can provide enhanced drug bioavailability, better drug-loading capacity, drug transport, polymer scaffold fabrication, vascular grafts, biosensing and diagnostic purposes, aseptic wound dressing, medical devices and others [31].

Hydrogels can provide a loose, porous structure and hydrated environment for culture cells, which, coupled with their resistance to disbanding, improves their fitness for different applications in emerging biomedical fields such as bio-sensing, drug delivery, tissue engineering, wound dressing, etc. [32,33,34].

**Absorption and adsorption**. Various ‘‘sorption’’ approaches (in the large sense of the definition) exist involving physical, chemical and biological interaction, and their combinations. Production of porous materials with high efficiency, selectivity, the ability to be reused multiple time, stability (better biodegradation) and cost-efficient production processes are important criteria in research, development and innovation in this field of gels science.

Non-modified cellulose materials have been traditionally used for absorption and adsorption purposes, however, their sorption capacity and selectivity are rather poor because of their intrinsic properties. To overcome the problem, cellulose derivatives are good quality candidates to be employed for sorption processes, in their native form or chemically modified (esterification, etherification, grafting of different moieties) to improve the desired properties for wastewaters, oil and organic fluid/solvents absorption, filtration, separation, precipitation, ion exchange, etc. [35,36].

## 5. Research, Development and Innovation in Cellulose-Based Gels

Cellulose-based gels research, development and innovation bring together various disciplines—chemistry, physics, materials science, environmental science, process engineering and medicine—to provide solutions for the development of innovative formulations and advanced materials with predictable components and controlled properties.

A survey of literature (Section 2, Section 3 and Section 4), previous and ongoing international research projects (as seen in Table 2) and recent patents (as seen in Table 3) on cellulose-based gels, both aerogels and hydrogels, reveals an important and prospective direction for their development—smart materials, such as biosensors, conducting electrolytes, printed electronics, etc. Known, traditional applications in pharmaceutical, medicine, the environment, and insulation, already participate in sustainable bio-economy and biorefinery approaches by obtaining specific products from natural polymer raw materials.

Fundamental and applied research on cellulose gels should be conducted to reveal the connection between cellulose chemistry and specific properties/abilities for specialized applications. Fundamental research involves the study of different forms of cellulose raw materials (native cellulose, cellulose fibers, cellulose derivatives, nanocellulose, nanofibers, nanofibrillated cellulose, etc.) and gel formation from them, while applied research consists of the development of gels with advanced desired properties.

Table 3 includes patents found in the Google Patent Database related to cellulose gels–aerogels and hydrogels from many different countries. The applications from the USA, China and Japan dominate the cellulose-based gels patents, but WIPO patents, European patents, Canadian and South Korean applications are also present. There are many cases in which the inventors came from different countries to those in which the application is filed, especially in the case of US patent applications.

The patents in cellulosic gels can be grouped into two categories: those involving gel synthesis approaches and those in related science and industrial applications. The main methods of gel manufacturing are chemical processes, followed by physical processes. Many preparation process details and related equipment are thought to be vital secrets by their inventors, so the information is very scarce, increasing the chances that patents in the field will. Patents on applications, improvements in performance and new materials will provide new means for innovative methods, devices and procedures and lead to new fields of research that will promote new frontlines in gel science. Preferred top tier domains, based on gels’ chemical, physical and mechanical properties, are conductive materials, pharmaceutical formulation, antiseptic applicator, smart packaging, magnetic composites, energy storage, etc.

Cellulose-based gels science and technology emerged decades ago, becoming scientifically relevant due to their low density, high specific area, non-toxicity and insulating properties. Today they are still used in various innovative materials because they provide possibilities for many new frontlines in today’s nanocellulose and nano-cellulose technology research, development and innovation [86].

Cellulose-based technology is truly multidisciplinary and interdisciplinary as it bridges many science and technology fields. Research projects and patents filed relating to cellulosic gels have increased rapidly, and this article only has cited a small part of them. The international research projects mentioned in this paper cover the development of numerous functional materials from cellulose, while the patents referred to in this paper cover a broad range of applications.

The main conclusion from research projects and patent applications is that the production of cellulose-based gels and their usefulness in diverse applications is an emerging area of interest for researchers and investors. We believe that more and more research projects and patents on cellulose-based gels will be published in the near future. However, there are still many challenges to overcome in order to improve industrial production, and scaling up production beyond the laboratory phase is absolutely necessary.

## 6. Concluding Remarks and Future Directions

Despite the acclaimed benefits and many gains and improvements, the large-scale, commercial production of cellulose-based gels still faces numerous and great challenges. Nevertheless, their technology readiness level (TRL) for the majority of the applications is still very low, despite the acclaimed successes achieved at the laboratory scale. More efforts should be made to increase the efficiency of raw materials processing and manufacturing processes. Furthermore, to advance the use of cellulose-based gels in new raw materials and novel extraction methods, improvements need to be made to reduce their costs. Moreover, specific equipment is needed to improve the technical feasibility and the efficiency of producing nano-scale cellulose needed in large quantities. The development of new products and processes needs to be achieved for the thorough utilization of components and to improve the entire process and the product economy. Following the joint efforts of researchers and stakeholders, it is reasonable to expect that cellulose-based gels will become an important material in meeting the ever-increasing property requirements.

It is clear that cellulose chemistry in gel formulation is fundamentally important for property shaping and for generating new functionalities. A profound understanding of cellulose chemistry in controlling the properties and applications is growing from studies of polymer and macromolecular chemistry; carbohydrate and polysaccharides chemistry; pulp and paper chemistry; and technology.

The accumulated knowledge in the field, even though it is not fully understood, will be valuable for researchers to continually design and fabricate novel gel formulations in a balanced manner. This, in turn, will allow further experimentation to improve our understanding of the roles of cellulose chemistry in rethinking the functionalities of cellulose-based gels and how to engineer new ones.

The future of cellulose-based gels appears to be bright and promises to be brighter because of the current attention on renewable raw materials, nontoxicity, biocompatibility, biodegradability, low costs and reducing our dependence on fossil raw materials to help reduce GHG emissions. Positive changes happen by starting with sustainable product development goals in all fields: technological, material, energy and economical, to devise alternative approaches to both traditional and value-added products and applications.

We are confident that, as additional modern and advanced techniques are used to open up new avenues for functional materials, we will see the materialization of many new products based on cellulose’s outstanding properties, accomplished through sustainable management essential for the benefit of everyone: local communities, businesses and the environment.

## Figures and Tables

**Table 1:** Properties/characteristics of the final produced aerogels and hydrogels.

Category	Property	Characteristics
Cellulose-based gels	Chemical	Radiation resistance, sunlight and UV resistance, weather (temperature, humidity, atmospheric pressure) resistance, recyclability
	Physical	Surface topology, texture, specific heat, density/porosity, thermal expansion, surface roughness, electrical conductivity, dielectric constant, thermal stability, color and esthetic
	Biological	Toxicity, biodegradability, biostability
	Mechanical/structural	Mechanical strength, Shear modulus, elastic modulus, fatigue strength, impact strength, creep resistance, yield strength, elongation to break,
	Technical	Fabrication costs, packaging, reproducibility, product quality, safety, life-cycle analysis

**Table 2:** International research projects on cellulose-based aerogels and hydrogels.

Acronym	Project Name	Cellulose-Based Molecule	Implementation Dates	Coordinator	Funding Programme	References
--	Polysaccharide upgrading via chemical and enzymatic modifications	Cellulose	1 December 1997–30 November 1999	University of Rome “La Sapienza”, Italy	FP4-TMR-Specific research and technological development programme in the field of the training and mobility of researchers, 1994–1998	[37]
--	The development of micro-biosensors for monitoring hazardous gases in the environment	Hydroxyethyl cellulose gel	1 November 1992–31 July 1995	University of Ioannina, Greece	FP3-ENV 1C-Specific research and technological development programme (EEC) in the field of the environment, 1990–1994	[38]
**--**	Aerocellulose and its carbon counterparts—porous, multifunctional nanomaterials from renewable resources	Cellulose	1 January 2004–31 December 2006	Lenzing Aktiengesellschaft, Austria	FP6-NMP-Nanotechnologies and nanosciences NMP-2002-3.4.2.3-2-New knowledge-based higher performance materials for macro-scale applications	[39]
AEROCOINS	Aerogel-Based Composite/Hybrid Nanomaterials for Cost-Effective Building Super-Insulation Systems	Nanofibrillated cellulose	16 June 2011–15 June 2015	Fundacion Tecnalia Research & Innovation, Spain	FP7-NMP-EeB.NMP.2010-1-New nanotechnology-based high-performance insulation systems for energy efficiency	[40]
AERoGELS	COST CA18125-Advanced Engineering and Research of aeroGels for Environment and Life Sciences	Cellulose	30 April 2019–26 February 2023	Universidad de Santiago de Compostela, Spain	COST (European Cooperation in Science and Technology) Action 2018	[41]
APACHE	Active & intelligent Packaging materials and display cases as a tool for preventive conservation of Cultural Heritage	Nanocellulose	1 January 2019–30 June 2022	Consorzio Interuniversitario Perlo Sviluppo Dei Sistemi A Grande Interfase, Italy	H2020-EU.2.1.3.-INDUSTRIAL LEADERSHIP-NMBP-33-2018-Innovative and affordable solutions for the preventive conservation of cultural heritage	[42]
BET-EU	Materials Synergy Integration for a Better Europe	Nanocellulose	1 January 2016–31 December 2018	Uninova-Instituto De Desenvolvimento De Novas Tecnologias–Associacao, Portugal	H2020-TWINN-2015-Twinning	[43]
BioELCell	Bioproducts Engineered from Lignocelluloses: from plants and upcycling to next-generation materials	Cellulose nanofibers	1 August 2018–31 July 2023	Aalto Korkeakoulusaatio SR, Finland	H2020-EU.1.1.-EXCELLENT SCIENCE- ERC-2017-ADG-ERC Advanced Grant	[44]
BIOGEL	Engineering responsive and biomimetic hydrogels for biomedical therapeutic and diagnostic applications	Nanocellulose	1 January 2015–31 December 2018	DWI Leibniz-Institut Fur Interaktive Materialien Ev, Germany	H2020-EU.1.3.1.-MSCA-ITN-2014-ETN-Marie Skłodowska-Curie Innovative Training Networks	[45]
BioMicroGels	Innovative environmentally-benign wastewater treatment reagents offering a step change in efficiency in the cleaning of water from oils and metal ions and in liquidation of emergency oil spills	Cellulose	1 August 2016–31 December 2016	BMG Intepco LTD, United Kingdom	H2020–MEInst-02- 2016-2017-Accelerating the uptake of nanotechnologies advanced materials or advanced manufacturing and processing technologies by SMEs	[46]
BIOSIC	Biopolymer-based Single-Ion Conducting Gel Polymer Electrolytes for Highly Performant and more Sustainable Batteries	Cellulose	1 September 2021–1 October 2023	Max–Planck-Gesellschaft Zur Forderung Der Wissenschaften EvGermany	H2020-EU.1.3.-EXCELLENT SCIENCE-Marie Skłodowska-Curie Actions	[47]
DRIVEN	Field-driven materials for functions, dissipation, and mimicking Pavlovian adaptation	Methylcellulose/Cellulose Nanocrystal	1 October 2017–30 September 2022	Aalto Korkeakoulusaatio SR Finland	H2020-EU.1.1.-EXCELLENT SCIENCE - ERC-2016-ADG-ERC Advanced Grant	[48]
INNPAPER	Innovative and Smart Printed Electronics based on Multifunctionalized Paper: from Smart Labelling to Point of Care Bioplatforms	Nanofibrillated Cellulose	1 January 2018–31 December 2021	Fundacion CIDETEC, Spain	H2020-EU.2.1.2.-INDUSTRIAL LEADERSHIP-PILOTS-05-2017-Paper-based electronics	[49]
H-House	Healthier Life with Eco-innovative Components for Housing Constructions	Cellulose	1 September 2013–31 August 2017	RISE CBI Betonginstitutet AB, Sweden	FP7-NMP-EeB.NMP.2013-2-Safe, energy-efficient and affordable new eco-innovative materials for building envelopes and/or partitions to provide a healthier indoor environment	[50]
MAEROSTRUC	Multicomponent Aerogels with Tailored Nano-, Micro- Macrostructure	Microcrystalline cellulose	1 March 2017–28 February 2022	Gottfried Wilhelm Leibniz Universitaet Hannover, Germany	H2020-EU.1.1.-EXCELLENT SCIENCE- ERC-2016-STG-ERC Starting Grant	[51]
NanoHybrids	New generation of nanoporous organic and hybrid aerogels for industrial applications: from the lab to pilot scale production	Cellulose	1 November 2015–30 April 2019	Technische Universitat Hamburg, Germany	H2020–NMP–PILOTS-2015-Manufacturing and control of nanoporous materials	[52]
NanoTextSurf	Nanotextured surfaces for membranes, protective textiles, friction pads and abrasive materials	Cellulose nanofibrils	1 November 2017–30 November 2020	Teknologian Tutkimuskeskus Vtt OY, Finland	H2020-EU.2.1.2.-INDUSTRIAL LEADERSHIP-PILOTS-03-2017-Pilot lines for manufacturing of nanotextured surfaces with mechanically enhanced properties	[53]
NewFUN	New era of printed paper electronics based on advanced functional cellulose	Cellulose nanocrystals	1 September 2015–31 May 2021	NOVA ID FCT-Associacao Para A Inovacao E Desenvolvimento Da FCT, Portugal	H2020-EU.1.1.-EXCELLENT SCIENCE-ERC-StG-2014-ERC Starting Grant	[54]
PlantEmulGel	Emulsions in Plant-based Edible Cellulose Microfibril Gels: Structure, Texture and Stability	Cellulose microfibril Gel	1 December 2018–30 November 2020	Unilever Innovation Centre Wageningen, Netherlands	H2020-EU.1.3-EXCELLENT SCIENCE-Marie Skłodowska-Curie Actions	[55]
PlantOleogels	Plant Particle-based Hybrid Bicontinuous Oleogels	Micro fibrillated cellulose	1 November 2018–31 October 2020	Unilever Innovation Centre Wageningen, Netherlands	H2020-EU.1.3-EXCELLENT SCIENCE-Marie Skłodowska-Curie Actions	[56]
SAM	Soft Artificial Muscles	Cellulose nanocrystals	1 March 2021–28 February 2023	Universite de Fribourg, Switzerland	H2020-EU.1.3.-EXCELLENT SCIENCE-Marie Skłodowska-Curie Actions	[57]
SYNERGY	Symbiosis for energy harvesting concepts for smart platforms on foils	Microcrystalline cellulose	1 October 2020–30 September 2023	UNINOVA-Instituto De Desenvolvimento De Novas Tecnologias-Associacao, Portugal	H2020-EU.4.b.-WIDESPREAD-05-2020-Twinning	[58]
WEARSENSNANO	Continuous monitoring of hypothermia in elderly people by the novel integrated wearable sensor system based on cellulose hydrogel and metallic nanowires	Cellulose	1 June 2021–31 May 2023	Aalto Korkeakoulusaatio SR, Finland	H2020-EU.1.3.2.-MSCA-IF-2020-Individual Fellowships	[59]
WoodNanoTech	Wood Nanotechnology for Multifunctional Structures	Nanocellulose	1 September 2017–31 August 2022	Kungliga Tekniska Hoegskolan, Sweden	H2020-EU.1.1.-EXCELLENT SCIENCE-ERC-2016-ADG-ERC Advanced Grant	[60]

**Table 3:** Recent selected patents on cellulose-based aerogels and hydrogels.

Reference Title	Publication Number	Raw Material	Inventors	Publication Date	References
A kind of preparation method of cellulose aerogels and its hybrid aerogel	CN105017555B China	Cellulose	Yu Jian, Ma Shurong, Mi Qinyong, Zhang Jun (Institute of Chemistry of CAS)	12 October 2018	[61]
A kind of preparation method of nanofibrils cellulose aerogel of ultralight, hydrophobic, high oil absorbency	CN103756006B China	Nanofibrils cellulose	Li Jian, Wancai Chao, Sun Qingfeng, Lu Yun, Gao Li, Kun Gan Wentao (Northeast Forestry University)	20 January 2016	[62]
A kind of preparation method of the elastic aerogel of multifunctional fiber element	CN105566673B China	Cellulose	Zhang Junping, Li Lingxiao, Li Bucheng (Lanzhou Institute of Chemical Physics LICP of CAS)	2 November 2018	[63]
Biodegradable single-phase cohesive hydrogel	CN101925348B China	Cellulose and cellulose derivative	Estelle Marie, Pirongueil Vitali (Laboratoires Vivacy SAS)	4 December 2013	[64]
Cellulose nanoparticle aerogels, hydrogels and organogels	US20130018112A1 United States	Cellulose nanoparticle	Wim Albert Wilfried Irene Thielemans, Rebecca Davies (University of Nottingham)	17 January 2013	[65]
Cellulose/black phosphorus nanosheet composite hydrogel and preparation method thereof	CN107936266B China	Cellulose	Zhang Han, Xing Chenyang, Chen Shiyou (Shenzhen University)	26 October 2021	[66]
Cellulose/two-dimensional layered material composite hydrogel and preparation method therefor	WO2019095751A1 WIPO	Cellulose	Zhang Han, Xing Chenyang, Chen Shiyou	23 May 2019	[67]
Cotton fiber dissolution and regeneration and 3D printing of cellulose-based conductive composites	US10311993B2 United States	Microcrystalline cellulose.	Noureddine Abidi, Yang Hu (Texas Tech University System)	4 June 2019	[68]
It is a kind of based on the dual network cellulose gel-based material being chemically and physically crosslinked	CN104448396B, China	Cellulose	Cai Jie, Li Kai, Zhao Dan, Zhang Lina	16 June 2017	[69]
Lithium-ion conductive material using bacterial cellulose organic gel, lithium-ion battery and bacterial cellulose airgel using the same	JP5110462B2, Japan	Bacterial cellulose	Shoichiro Yano, Takashi Sawaguchi, Shunki Hagihara, Hideaki Maeda, Ei Nakajima, Ichihiro Sasaki	26 December 2012	[70]
Manufacturing method of cellulose aerogel membrane	KR101494641B1 South Korea	Cellulose	Kim Chang-yeol, Go Eun-byeol	24 February 2015	[71]
Medical hydrogel	CN110072567B, China	Nanofibrillar cellulose	K. Luko M. Nopening (UPM Kymmene Oy)	12 April 2022	[72]
Method and apparatus for processing fibril cellulose and fibril cellulose product	EP2815026B1, European Patent Office	Nanofibrillar cellulose	Antti Laukkanen, Markus Nuopponen (UPM Kymmene Oy)	16 June 2021	[73]
Method for preparing amorphous cellulose aerogel with ionic liquid	CN102443188B China	Cellulose	Lu Yun Sun Qingfeng Liu Yixing Yu Haipeng Yang Dongjiang (Northeast Forestry University)	13 March 2013	[74]
Method for processing nanofibrillar cellulose	CA2824125C, Canada	Nanofibrillar cellulose	Antti Laukkanen, Jan-Erik Teirfolk, Markus Nuopponen (UPM Kymmene Oy)	7 May 2019	[75]
Method for producing a gel-based composite material	JP6224175B2, Japan	Cellulose fibers	Patrick A, Sea, Gain Michelle, Cienkar Lambsey, Subra Manian Joachim, Ciel Kotup	1 November 2017	[76]
Method for producing nanofibril cellulose gel	JP6698236B1, Japan	Nanofibril Cellulosic fibers	Patrick, A, Sea, Gain Joachim, Ciel Kotup Daniel, Gantenbain Michel Cienker	27 May 2020	[77]
Method for producing nanofibrillar cellulose and nanofibrillar cellulose product	US11274396B2 United States	Nanofibrillar cellulose	Markus Nuopponen, Juha Tamper, Isko Kajanto (UPM Kymmene Oy)	15 March 2022	[78]
Methods for Making Structured Materials Using Nanofibril Cellulose Gel	JP7033105B2, Japan	Cellulose fibers	Patrick A, Sea, Gain Michelle, Cienkar Lambsey, Subra Manian Joachim, Ciel Kotup	9 March 2022	[79]
Nanofibrillar cellulose composition	US10729804B2 United States	Nanofibrillar cellulose	Marjo Yliperttula, Patrick Lauren, Petter Somersalo, Yanru Lou (UPM Kymmene Oy)	4 August 2020	[80]
Nanofibrillar polysaccharide for use in the control and prevention of contraction and scarring	EP2958599B1 European Patent Office	Nanofibrillar cellulose	Antti Laukkanen, Esko Kankuri, Kristo Nuuutila (UPM Kymmene Oy)	16 November 2016	[81]
Porous cellulose gel, method for producing the same, and use thereof	US9446382B2, United States	Crosslinked spherical crystalline cellulose particles	Yasuto Umeda, Yasuo Matsumoto, Masami Shiina, Masami Todokoro, Yoshihiro Matsumoto (JNC Corp)	20 September 2016	[82]
Regenerated cellulose film, functional film and preparation method thereof	EP3064534B1 European Patent Office	Regenerated cellulose	Jun Zhang, Xiaoyu Zhang, Jian Yu, Ruifeng Li, Jin Wu, Yugang GAO, Jinming Zhang, Jinjiang QIU (Inst of Chemistry Chinese Academic of Sciences, Institute of Chemistry of CAS)	20 October 2021	[83]
Transparent cellulose hydrogel and production process thereof	US5962005A United States	Regenerated cellulose	Hiroshi Saga, Hidenao Saito (Rengo Co Ltd.)	10 May 1999	[84]
Wound healing compositions comprising biocompatible cellulose hydrogel membranes and methods of use thereof	US9314531B2 United States	Microcrystalline cellulose and bacterial cellulose,	Morgana M. Trexler, Jennifer H. Elisseeff, Daniel Mulreany, Qiongyu Guo, Jennifer L. Breidenich, Jeffrey P. Maranchi, Jenna L. Graham, Julia B. Patrone, Marcia W. Patchan, Xiomara Calderon-Colon (Johns Hopkins University)	19 April 2016	[85]

## Data Availability

Not applicable.

## Appendix A

The nomenclature used to describe observations of cellulose-based gels, aerogels and hydrogels often varies considerably among researchers, laboratories, articles and textbooks in the fields of wood/cellulose science and technology. Standard scientific, technical and chemical dictionaries often differ in their definitions of commonly used term in gel material research. This lack of a common terminology leads to misunderstanding and ambiguity when communicating scientific findings, and can also be a particular drawback for some or a dishonest benefit for others in submitting manuscripts to journals worldwide. Therefore, there is a recognized need for a universal nomenclature to be used when describing such observations and for this, the current review used the terms defined according to IUPAC Gold Book [1] and current terminology in polymer chemistry [2]. Other alternative definitions or terms found in the literature, such as aerocellulose, bio-aerogel, all-cellulosegels, xerogels and cryogels, although correct in their meaning, were not used.

aquagel = hydrogel in which the network component is a colloidal network.

aerogel = gel comprised of a microporous solid in which the dispersed phase is a gas.

Note:

Microporous silica, microporous glass and zeolites are common examples of aerogels.

gel = non-fluid colloidal network or polymer network that is expanded throughout its whole volume by a fluid.

Notes:

1. A gel has a finite, usually rather small, yield stress.

2. A gel can contain:

(a) a covalent polymer network, e.g., a network formed by crosslinking polymer chains or by non-linear polymerization;
(b) a polymer network formed through the physical aggregation of polymer chains, caused by hydrogen bonds, crystallization, helix formation, complexation, etc., that results in regions of local order acting as the network junction points. The resulting swollen network may be termed a thermoreversible gel if the regions of local order are thermally reversible;
(c) a polymer network formed through glassy junction points, e.g., one based on block copolymers. If the junction points are thermally reversible glassy domains, the resulting swollen network may also be termed a thermoreversible gel;
(d) lamellar structures including mesophases, e.g., soap gels, phospholipids and clays;
(e) particulate disordered structures, e.g., a flocculent precipitate usually consisting of particles with large geometrical anisotropy, such as in V_2_O_5_ gels and globular or fibrillar protein gels.

3. Corrected from the previous definition where the definition is via the property identified in Note 1 (above) rather than of the structural characteristics that describe a gel.

hydrogel = gel in which the swelling agent is water.

Notes:

1. The network component of a hydrogel is usually a polymer network.

2. A hydrogel in which the network component is a colloidal network may be referred to as an aquagel.
